# Association between environmental gradient of anthropization and phenotypic plasticity in two species of triatomines

**DOI:** 10.1186/s13071-024-06258-w

**Published:** 2024-04-02

**Authors:** Federico G. Fiad, Miriam Cardozo, Julieta Nattero, Gisel V. Gigena, David E. Gorla, Claudia S. Rodríguez

**Affiliations:** 1 Cátedra de Morfología Animal, Facultad de Ciencias Exactas, Físicas y Naturales, Universidad Nacional de Córdoba, Córdoba, Argentina; 2 Instituto de Investigaciones Biológicas y Tecnológicas (IIBYT), CONICET, Córdoba, Argentina; 3 Cátedra de Introducción a la Biología, Facultad de Ciencias Exactas, Físicas y Naturales, Universidad Nacional de Córdoba, Córdoba, Argentina; 4 Departamento de Ecología Genética y Evolución, Laboratorio de Eco-Epidemiología, Facultad de Ciencias Exactas y Naturales, Universidad de Buenos Aires, Ciudad Autónoma de Buenos Aires, Argentina; 5 Instituto de Ecología, Genética y Evolución (IEGEBA), CONICET, Ciudad Autónoma de Buenos Aires, Argentina; 6 Instituto de Diversidad y Ecología Animal (IDEA), CONICET, Universidad Nacional de Córdoba, Córdoba, Argentina

**Keywords:** Chagas disease, Head, Land cover changes, *Triatoma garciabesi*, *Triatoma guasayana*, Wing

## Abstract

**Background:**

*Triatoma garciabesi* and *T. guasayana* are considered secondary vectors of *Trypanosoma cruzi* and frequently invade rural houses in central Argentina. Wing and head structures determine the ability of triatomines to disperse. Environmental changes exert selective pressures on populations of both species, promoting changes in these structures that could have consequences for flight dispersal. The aim of this study was to investigate the relationship between a gradient of anthropization and phenotypic plasticity in flight-related traits.

**Methods:**

The research was carried out in Cruz del Eje and Ischilín departments (Córdoba, Argentina) and included 423 individuals of the two species of triatomines. To measure the degree of anthropization, a thematic map was constructed using supervised classification, from which seven landscapes were selected, and nine landscape metrics were extracted and used in a hierarchical analysis. To determine the flight capacity and the invasion of dwellings at different levels of anthropization for both species, entomological indices were calculated. Digital images of the body, head and wings were used to measure linear and geometric morphometric variables related to flight dispersion. One-way ANOVA and canonical variate analysis (CVA) were used to analyze differences in size and shape between levels of anthropization. Procrustes variance of shape was calculated to analyze differences in phenotypic variation in heads and wings.

**Results:**

Hierarchical analysis was used to classify the landscapes into three levels of anthropization: high, intermediate and low. The dispersal index for both species yielded similar results across the anthropization gradient. However, in less anthropized landscapes, the density index was higher for *T. garciabesi*. Additionally, in highly anthropized landscapes, females and males of both species exhibited reduced numbers. Regarding phenotypic changes, the size of body, head and wings of *T. garciabesi* captured in the most anthropized landscapes was greater than for those captured in less anthropized landscapes. No differences in body size were observed in *T. guasayana* collected in the different landscapes. However, males from highly anthropized landscapes had smaller heads and wings than those captured in less anthropized landscapes. Both wing and head shapes varied between less and more anthropogenic environments in both species.

**Conclusions:**

Results of the study indicate that the flight-dispersal characteristics of *T. garciabesi* and *T. guasayana* changed in response to varying degrees of anthropization.

**Graphical Abstract:**

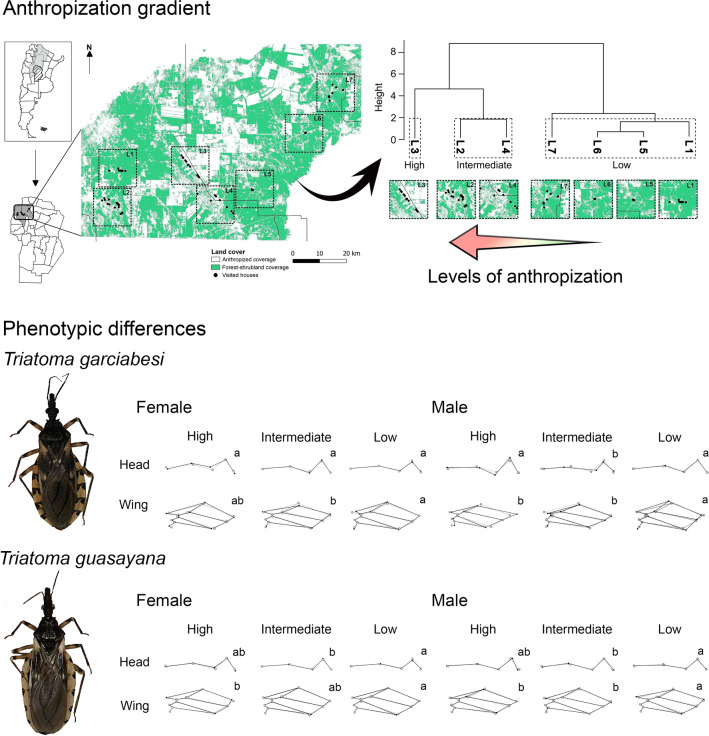

**Supplementary Information:**

The online version contains supplementary material available at 10.1186/s13071-024-06258-w.

## Background

*Triatoma garciabesi* and *T. guasayana* (Hemiptera: Reduviidae, Triatominae) are considered secondary vectors of *Trypanosoma cruzi*, the causative agent of Chagas disease. These insects maintain the transmission cycles of the parasite in the wild, and thus could therefore link the transmission of the parasite from the wild to the domestic environment [1, 2]. Both species are distributed throughout Argentina, Bolivia and Paraguay [3, 4]. *Triatoma garciabesi* is a bird-associated arboreal species that inhabits the loose bark of *Prosopis* sp. in high population densities throughout the year [5]. *Triatoma guasayana* is a ground-dwelling species associated with mammals. It typically inhabits dry cacti, fallen logs and bromeliads. The population density of this species decreases during the winter season [6, 7]. Several studies have reported that both species frequently invade rural houses in Argentina during the summer season in the western Chaco Seco ecoregion [5, 8–12]. Degradation of natural environments due to advancing anthropogenic activities may indirectly affect triatomine populations, leading to their dispersal into artificial ecotopes [13]. Changes in the local environment, including decreases in bird and mammal populations that serve as food sources, and the loss of natural habitats, may exert selective pressures on flight-related traits that enhance mobility in a dynamic environment [14, 15].

The Gran Chaco is an ecologically diverse ecoregion that spans Argentina (62.19%), Paraguay (25.43%), Bolivia (11.61%) and Brazil (0.77%). In recent decades, agriculture has been extensively expanded in this region [16], resulting in land use changes due to agriculture and cattle raising practices with significant impacts on the landscape [17, 18]. In the northwestern region of Córdoba (Argentina), the percentage of anthropogenic landscape increased from 16% in 1979 to 30% in 2010 [17]. Analyzing the impact of land use change on vegetation cover and the environment is essential for the implementation of sustainable land management practices. The anthropogenic gradient delineates the process of shifting from natural landscape with consistent vegetation coverage to semi-natural landscape that encompasses both natural and anthropogenically influenced land cover. This progression conclusively culminates in an anthropized landscape, where cultural land cover is predominant and natural vegetation coverage is relatively low [19, 20]. This process affects the stability of the local flora and fauna generating strong environmental pressures on the organisms [21, 22]. Besides, insufficient connectivity and environmental heterogeneity have a negative impact on individuals and could affect phenotypic variation in dispersal-associated traits at the landscape scale [15].

Phenotypic variations in insects are influenced by developmental conditions during juvenile stages and shaped by environmental pressures, potentially enhancing dispersal processes [23]. The evolution of dispersive strategies and changes in dispersive phenotypes are determined by the balance between costs and benefits that influence reproductive outcomes [15, 24]. The decrease in the flight musculature in triatomines may result in the allocation of energetic resources to reproduction, leading to higher reproductive capacity at the cost of lower dispersive capacity [25, 26]. During dispersal events, heads and wings play critical roles as fundamental structures. It has been proposed that narrow heads, with the associated development of compound eyes, have been proposed to be correlated with morphotypes that allow improved flight in triatomines [27–29]. Furthermore, small changes in wing shape may significantly affect aerodynamic performance [30]. Insects with elongated, slender wings are compatible with fast, straight long-distance flight, whereas short, broad wings are optimal for slow, agile short-distance flight [31, 32]. The wings of most insects have zones tailored for deformation and other areas that help to limit deformations and prevent structural damage [33]. The hemelytra of Heteroptera have two distinct zones. The anterior sclerotic zone provides support and regulates wing deformation, while the distal membranous zone is susceptible to deformation. According to Wootton [34], the latter zone is more aerodynamically efficient. An increase in size and area of this zone could therefore indicate improved flight performance.

Long-distance movements across degraded landscapes may require trait adaptations that lead to improved movement efficiency [15]. Consequently, anthropogenic changes may act as an environmental selective pressure that shapes phenotypic adaptations associated with flight traits. The aim of this study was to analyze flight-dispersal traits and their relationship with increasing anthropization in *T. garciabesi* and *T. guasayana*. These two species, chosen for their differing life strategies and wild habits, provide a valuable framework for studying the impact of ecological factors on triatomine distribution and disease transmission dynamics in these landscapes. Furthermore, their abundance and accessibility in the study area enable comprehensive field surveys in anthropized environments. In this study, *T. garciabesi* was more frequent where there was an intermediate vegetation cover near the house and within a 1000-mts circle around the house. *Triatoma guasayana* was more frequent with low vegetation cover near the house (≤ 100 mts) and intermediate vegetation cover within a circle of 1000 mts around the house. This might suggest isolated populations of each species in rural anthropized environments. Additionally, different entomological indices were studied at each anthropization level as estimators of flight and residential invasion capacity, and the analysis was performed between species and sexes.

## Methods

### Study area and insect collection

The study was conducted at the beginning (October-December 2018, 2019) and at the end (February, March 2019 and 2020) of the warm season 2018 and March 2020, using the methodology presented by Cardozo et al. [35]. A Garmin Etrex 30 GPS device was used to georeferenced and mark a total of 131 dwellings in 14 rural communities in the departments of Cruz del Eje and Ischilín, located in the northwestern of Córdoba Province, central Argentina. Residents of selected households were provided with small plastic bags to collect triatomines from their dwellings. Triatomines were collected after 15 days and then taxonomically identified, sexed, photographed, dissected and preserved in 70% alcohol. The taxonomic identification of the collected triatomines was carried out by observing their external morphology using the keys of Lent and Wygodzinsky [36] and Jurberg et al. [37]. To reduce seasonal variation, only insects found at the beginning of the summer season were analyzed [38]. A total of 104 specimens of *T. garciabesi* (53 females and 51 males) and 312 specimens of *T. guasayana* (210 females and 102 males), were studied. Both sexes were considered separately in both species due to studies suggesting the presence of sexual dimorphism in triatomines [27, 28, 36, 39].

### Characterization of the anthropization gradient

**Fig. 1:**
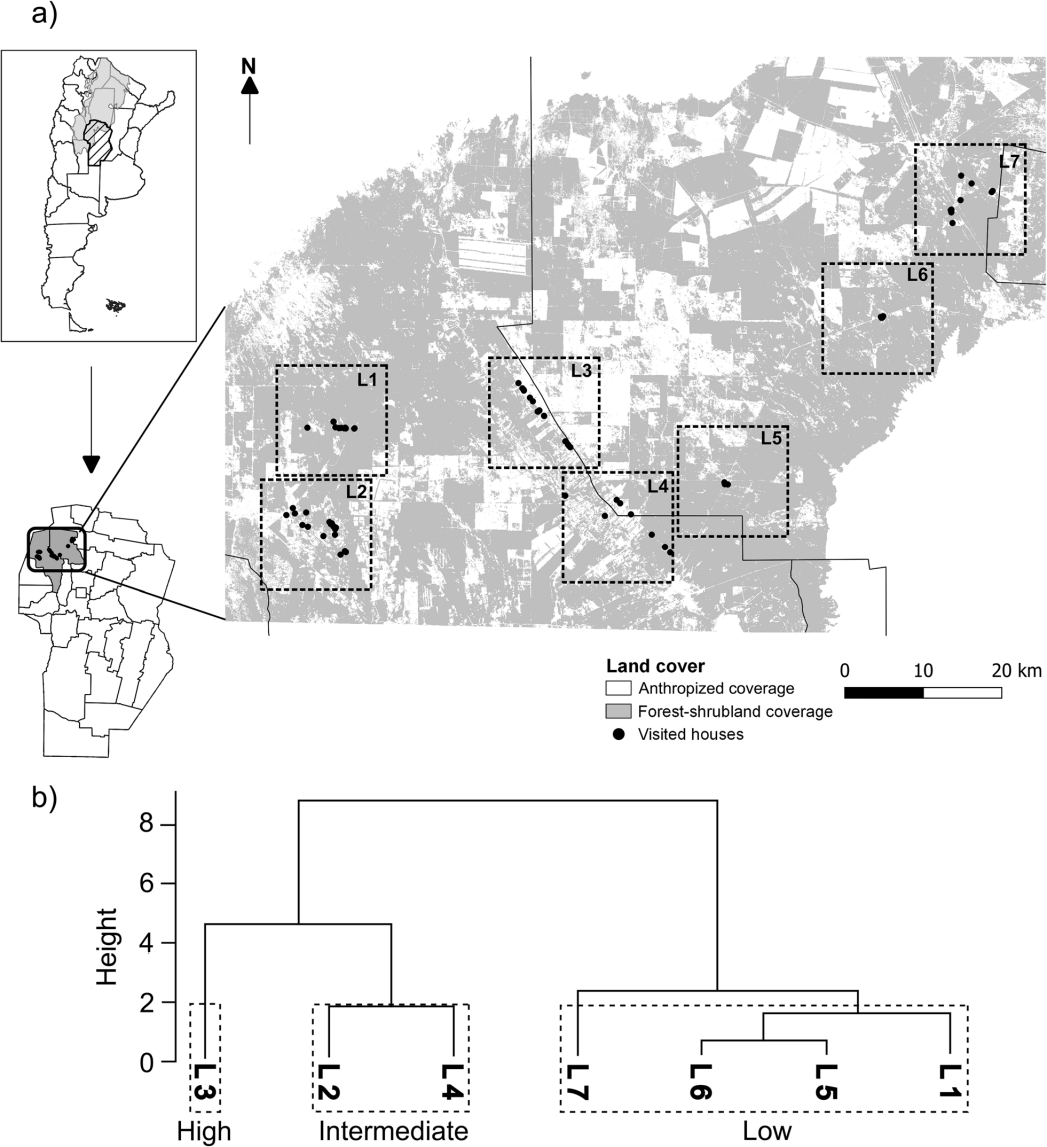
**a** Thematic map displaying natural features (forests, shrubland and water) and anthropogenic features (bare soil, managed grassland and crops). Black dots indicate the location of dwellings visited during the sampling campaign conducted from 2018 to 2020. Dotted line squares illustrate the boundaries of the seven selected landscapes. **b** Additionally, a landscape taxonomy dendrogram plotted from similarity analysis using Ward’s method incorporates nine landscape metrics, accounting for composition and configuration. High, intermediate and low are indicated in the boxes. *L1* Cachiyuyo, *L2* Las Abras-Iglesia Vieja, *L3* Hormigueros-Chañaritos, *L4* El Simbolar-El Tropiezo-El Barreal-Palo Parado, *L5* Huascha, *L6* Jaime Peter, *L7* San Nicolás de las Chacras-Los Churquis-El Bañado

To characterize the anthropization gradient, 410 Sentinel 2 Level 2A images with atmospheric bottom correction from the Copernicus Image Repository (ESA) [40] were utilized for the Cruz del Eje and Ischilín areas. Atmospheric correction at ground level was performed using dark subtraction and radiometric calibration on the satellite images, albeit these processes were conducted by ESA [41]. These images spanned from January 1, 2018, to December 31, 2020. To mitigate the impact of cloud cover, a cloud and cirrus filter was applied to the scenes obtained from the selection to create a layer mask. Subsequently, this layer mask was applied to the images affected by cloud cover, particularly during the rainiest months (December, January, February), ensuring the preservation of pixel information. Land cover types were identified by supervised classification using 36 transects drawn from 18 residences selected based on their landscape characteristics to serve as ground truth sites [42]. The kappa coefficient and precision were calculated via a confusion matrix, which collected conflicts between categories, to define the quality of the thematic map obtained. The thematic map classes were divided into two categories: natural and artificial. The natural category grouped closed and open forest, closed and open shrubland, and water classes, while the artificial category involved unvegetated soil, managed pastures and crops. To examine the anthropization gradient, seven landscapes measuring 196 km^2^ with varying levels of natural coverage were chosen for analysis near each location (Fig. 1a). The database, codes and obtaining the thematic map from supervised classification are available on the online repository [43]. Imaging, pre-processing and analysis were performed using the Google Earth Engine (GEE) platform, while visualization and post-processing were performed in QGIS v3.26.2. Landscape metrics for configuration and composition were derived using the FRAGSTATS v4.2 software (Additional file 1: Table S1). A hierarchical clustering analysis using Ward’s method was performed on the nine-landscape metrics [44] to categorize landscapes based on their anthropization gradient.

### Comparison of entomological indexes and phenotypes along the anthropization gradient

Following the methodology of Silveira et al. [45], we calculated entomological indices per species to assess the flight capability and invasion at the different levels of anthropization. The dispersal index was calculated by dividing the number of positive dwellings by the total number of dwellings visited and multiplying the result by 100. The density index was calculated by dividing the number of adult triatomines by the number of visited dwellings. The sex ratio was determined by dividing the number of male triatomines by the number of female triatomines and multiplying the outcome by 100.

**Fig. 2:**
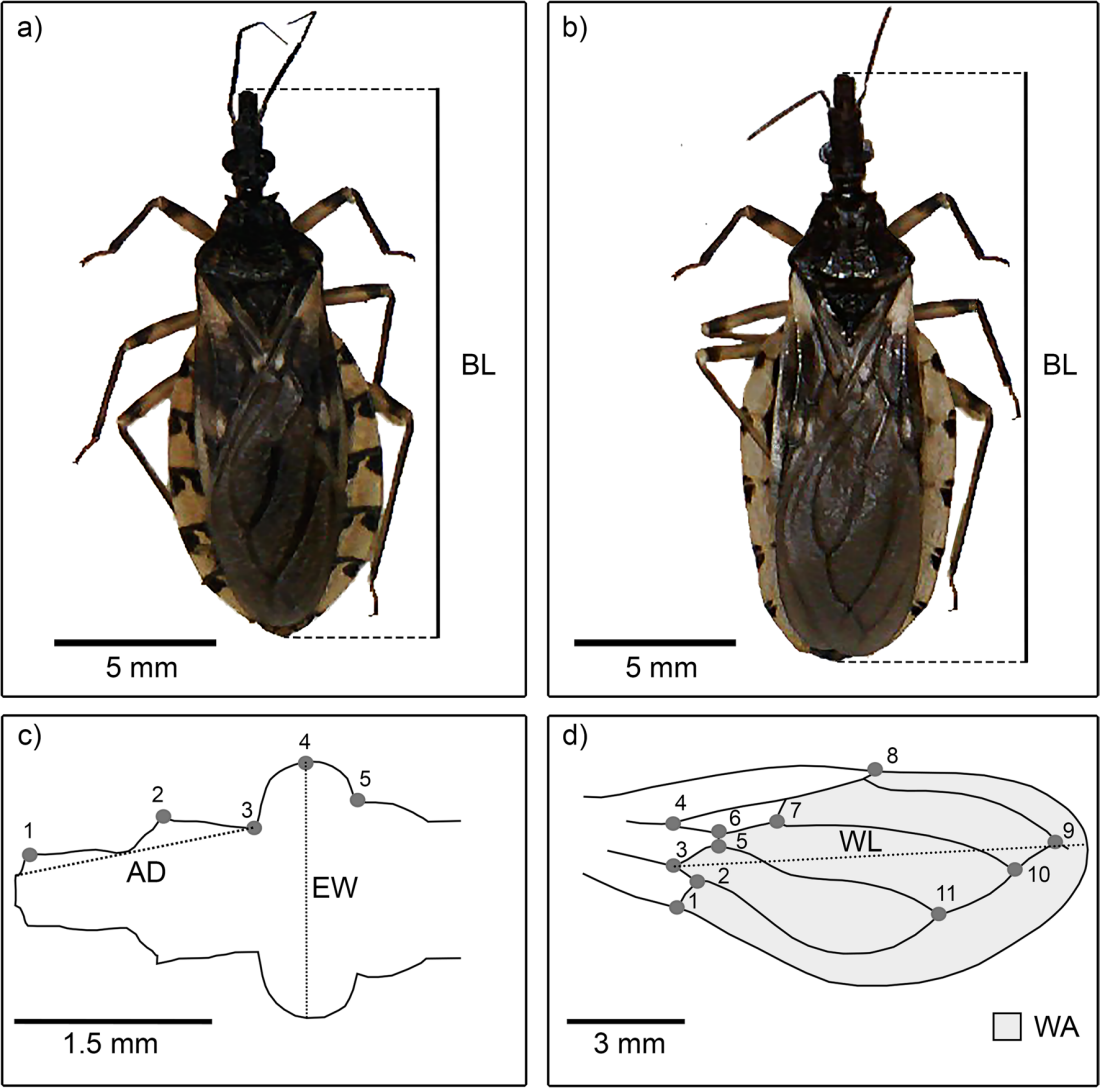
Specimens of *Triatoma garciabesi* (**a**) and *T. guasayana* (**b**). *BL* body length. Linear measurements and morphological landmarks type II on the head (**c**) and type I on the wings (**d**). AD (anteocular distance in mm), EW (maximum distance between eyes in mm), WL (maximum wing length of the membranous region in mm) and WA (area of the wing membranous region in mm^2^)

To compare the phenotypic sizes in relation to the anthropization gradient, digital photographs of the body, head and wings of each specimen were taken with a reference scale using an Olympus VG 160 camera attached to a Zeiss SV 11 stereo microscope. Body, head and wing measurements in millimeters were obtained using the free ImageJ software, version 1.53t (https://imagej.nih.gov/). Body length (BL) was measured from the clipeous to the last abdominal segment (Fig. 2a, b). Linear measurements of the head were taken, including the distance between the eyes (EW) and the distance from the eyes to the base of the head (AD) (Fig. 2c). Wing measurements were also recorded, including the maximum wing length (WL) and the area of the membranous region (WA) (Fig. 2d).

To investigate phenotypic shape differences in structures involved in flight dispersal along the anthropization gradient, we identified 5 type II morphological landmarks in the head of the right half [27, 46] (Fig. 2c) and 11 type I morphological landmarks in the right wing (Fig. 2d). The digitization of the geometric coordinates was carried out using tpsDIG v2.32 [47]. To evaluate the digitization error, the landmarking process was repeated. Damaged specimens that did not allow identification of morphologic landmarks were not included. To calculate the morphological variation in the anthropization gradient, we used the formula ${\text{V}} = \sum\nolimits_{{{\text{j}} = 1}}^{{\text{N}}} {{{{\text{D}}_{{\text{j}}}^{2} } \mathord{\left/ {\vphantom {{{\text{D}}_{{\text{j}}}^{2} } {\left( {{\text{N}} - 1} \right)}}} \right. \kern-0pt} {\left( {{\text{N}} - 1} \right)}}}$, where D_j_^2^ represents the Procrustes distance between the shape of specimen j and the overall mean shape for each population, N, represents the levels of anthropization [48].

### Statistical analyzes

Entomological indices were compared between anthropization levels using chi-squared analysis to assess flight and invasion by species and sex. In addition, ANOVA and Tukey post-hoc tests were used to analyze potential differences in size phenotypes for variables with normal distributions and Kruskal-Wallis/Dunn post-hoc tests for variables without normal distributions (Shapiro-Wilks test with a significance level of 0.05). To obtain shape variables and centroid sizes, generalized Procrustes analyses were performed by species and sex for heads and wings of Type II and Type I morphological landmarks, respectively. To analyze configuration differences between anthropization levels, canonical variable analysis (CVA) was performed, and group means were compared via permutation analysis. To compare the size differences of structures associated with the three levels of anthropization, centroid size (CS) was compared between anthropization levels. In addition, we assessed the changes in phenotypic variation along the anthropization gradient; pairwise comparisons to identify differences among the anthropization levels were conducted. We evaluated the allometric component by performing multivariate regressions with shape as the dependent variable and the logarithm of CS as the independent variable. To examine the correlation between changes in shape of structures and geographic distances across various levels of anthropization, we conducted Mantel tests. Results with a *P* < 0.05 were considered significant. Geometric morphometric analyses were performed using the *geomorph* [49] and *Morpho* [50] software packages in the R programming language, with a total of 10,000 permutations for all analyses.

## Results

### Gradient of anthropization in the study area

Thematic map with eight coverage classes was obtained (Fig. 1a; Additional file 2: Table S2) [43]. The algorithm correctly classified the classes with an overall accuracy of 99.31% and a corrected kappa index of 0.9915 (Additional file 2: Table S2).

Hierarchical similarity analysis allowed classifying the seven landscapes (L1–7) into three levels of anthropization: high (L3), intermediate (L2 and L4) and low (L1, L5, L6 and L7), with good support in the groupings (*W* = 0.792) (Fig. 1b). The coverage areas and percentages of natural vegetation, as well as patch sizes, showed a marked decline from the low anthropization level to the high level. Conversely, the number of patches and patch edge area increased, signaling a higher degree of fragmentation at the high anthropization level. The patch distribution across the landscape showed homogeneity at the low and intermediate levels of anthropization, revealing good connectivity between the patches. However, at the high level of anthropization, the patch distribution was more dispersed.

### Entomological indices and phenotypic differences related to anthropogenic gradient

**Fig. 3:**
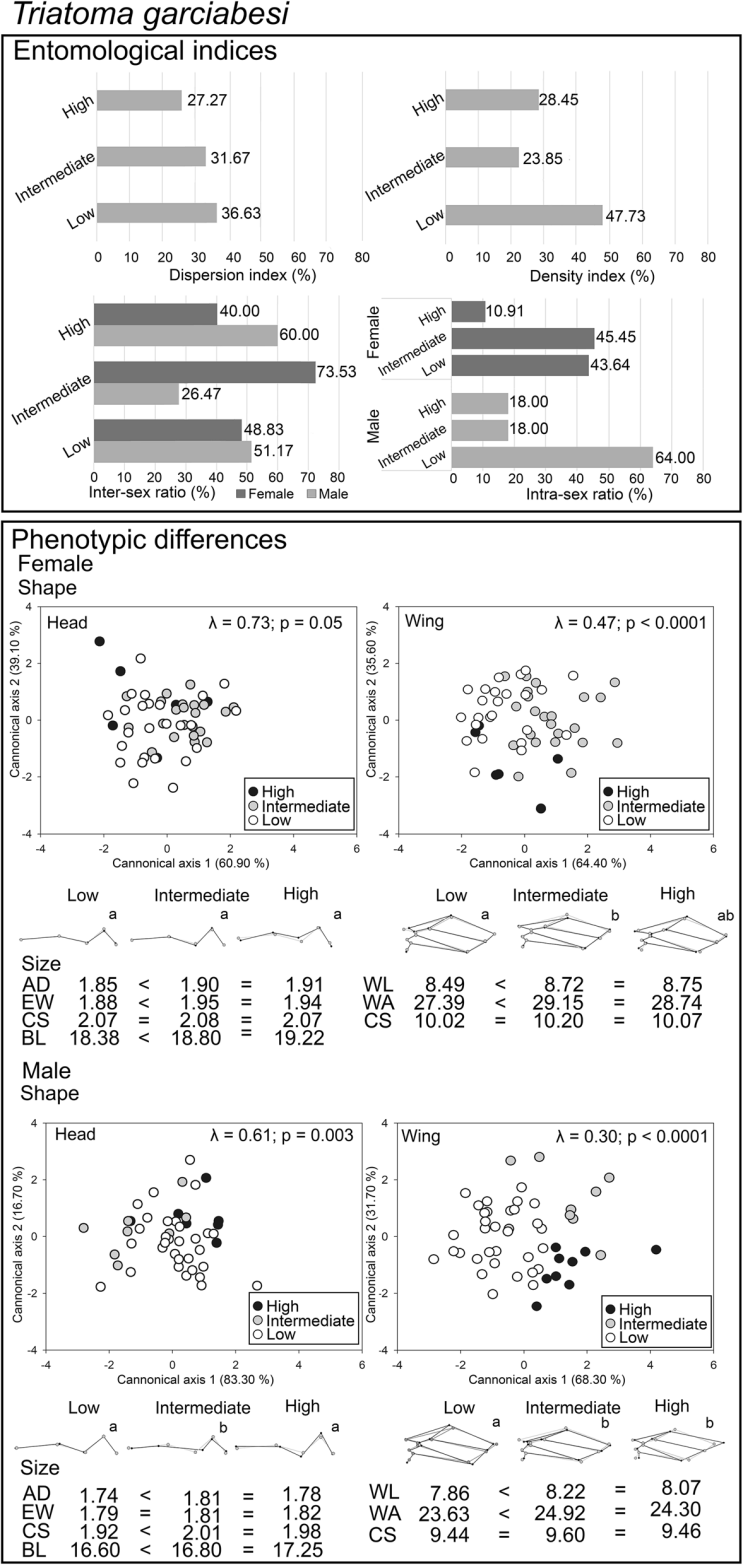
Results were obtained for entomological indices and phenotypic differentiation of individuals belonging to *Triatoma garciabesi* at three levels of anthropization: high, intermediate and low. The graph displays percentages for each entomological index, shape and mean size calculated for each anthropization level, utilizing a scale factor of 0.15 Procrustes distance units for improved visualization. Different letters indicate statistically significant differences between groups for the same reference at *P* < 0.05. Scatterplot depicting the results of the Canonical Variate Analysis (CVA) for head and wing measurements across three levels of anthropization. Wilk’s lambda values and their associated *P*-values for each CVA are reported. *BL* body length, *AD* anteocular distance, *EW* maximum interocular distance, *WL* wing length of the membranous region, *WA* area of the membranous region, *CS* centroid size of the right side of the head and wing. Inter-sex comparisons were made between females and males within each anthropization level. Intra-sex comparisons were made between females:females and males:males between anthropization levels

**Fig. 4:**
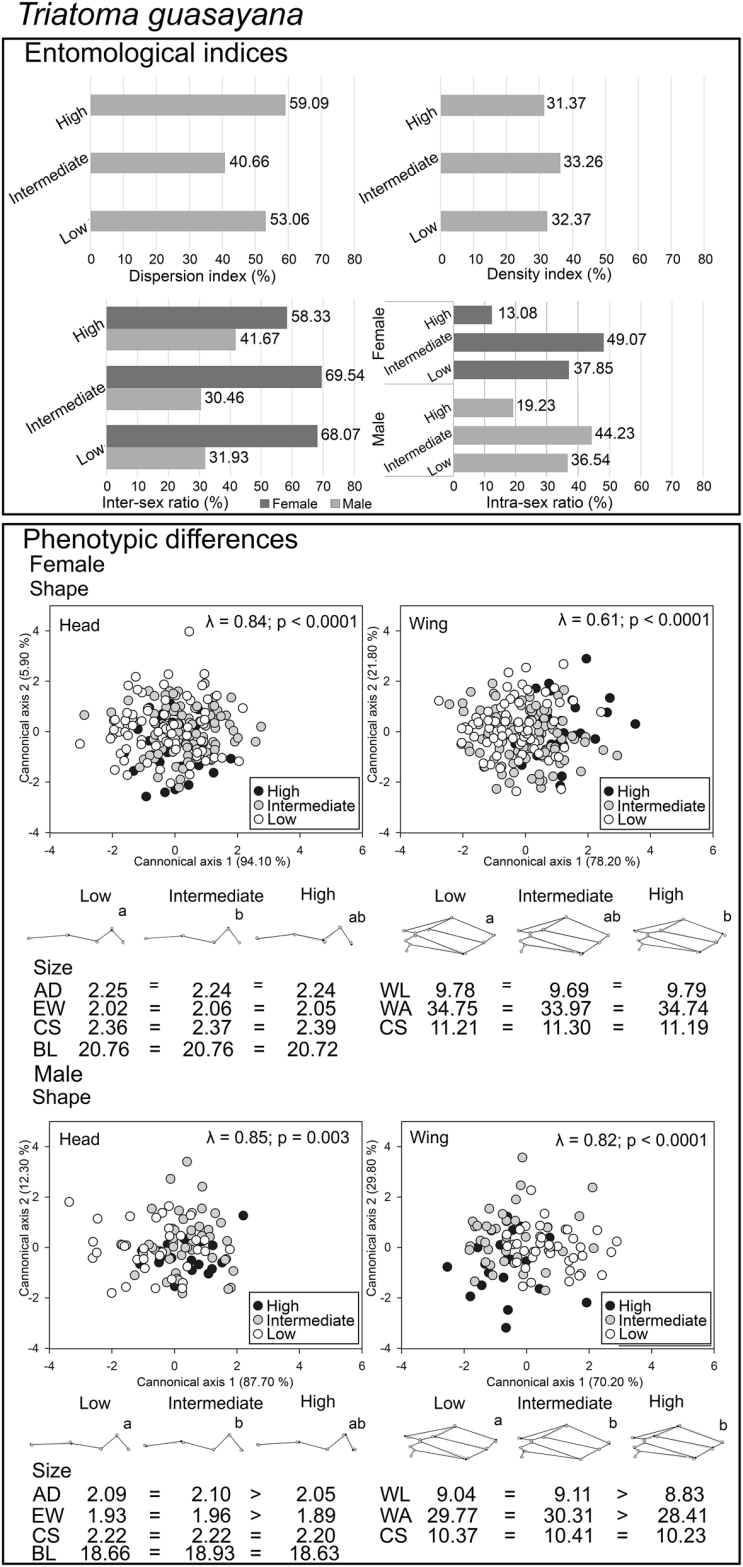
Results were obtained for entomological indices and phenotypic differentiation of individuals belonging to *Triatoma guasayana* at three levels of anthropization: high, intermediate and low. The graph displays percentages for each entomological index, shape and mean size calculated for each anthropization level, utilizing a scale factor of 0.15 Procrustes distance units for improved visualization. Different letters indicate statistically significant differences between groups for the same reference at *P* < 0.05. Scatterplot depicting the results of the Canonical Variate Analysis (CVA) for head and wing measurements across three levels of anthropization. Wilk’s lambda values and their associated *P*-values for each CVA are reported. *BL* body length, *AD* anteocular distance, *EW* maximum interocular distance, *WL* wing length of the membranous region, *WA* area of the membranous region, *CS* centroid size of the right side of the head and wing. Inter-sex comparisons were made between females and males within each anthropization level. Intra-sex comparisons were made between females:females and males:males between anthropization levels

*Triatoma garciabesi* had 24.82% (105/423) of the triatomine specimens captured while *T. guasayana* represented 75.18% (318/423). The density index for *T. garciabesi* (*χ*^2^ = 9.60; *df* = 2; *P* = 0.008) (Fig. 3) was higher in less anthropized landscapes, while for *T. guasayana* (*χ*^2^ = 0.82; *df* = 2; *P* = 0.54) (Fig. 4) it was similar between levels of anthropization. The sex ratio of both species indicated differences in intermediate levels of anthropization and in low levels of anthropization for *T. guasayana*, where more females were dispersed. The proportion of male and female individuals of these species decreased in highly anthropized landscapes (Figs. 3 and 4).

Individuals of *T. garciabesi* captured in the least anthropized landscapes had smaller sizes than those captured in the most anthropized landscapes (Fig. 3; Additional file 3: Table S3a). No association was found between centroid size and the shape (allometric effects) for either wings or heads across any of the species studied (Additional file 5: Table S5). Procrustes distances of the head and wing shape showed significant differences between less anthropized and increasingly anthropized landscapes (Table 1). The antennal tubercles showed greater expansion, and the compound eyes showed less convexity. Wing shape in landscapes with low levels of anthropization was rounded in the distal region of the hemelytra, whereas in landscapes with increasing anthropization, the wings had a laterally narrow shape with the wing tip ending in a point (Fig. 3). In contrast, *T. guasayana* from landscapes with high anthropogenic impact had smaller heads and wings compared to the other anthropization levels (Fig. 4; Additional file 3: Table S3b). Procrustes distances of head and wing shape showed significant differences between low and increasing anthropization (Table 1). Head shape showed greater expansion of antennal tubercles and greater convexity of compound eyes in insects at intermediate levels of anthropization (Fig. 4). In males, wings in landscapes with low levels of anthropization had a broad distal end and wing base, whereas those in landscapes with the highest anthropization level had a thin distal end with elongated upper ends ending in a tip. Female wings in landscapes with high levels of anthropization were broad and rounded in the distal region (Fig. 4). The number of accurately assigned individuals was greater for *T. garciabesi* compared to *T. guasayana* (Additional file 4: Table S4). No significant associations were observed between geographic distances and head configuration (Additional file 5: Table S5).

**Table 1:** Procrustes distances between mean values of low, intermediate and high levels of anthropization for populations of *Triatoma garciabesi* and *T. guasayana* performed for wing and head shape variation

Species	Sex	Comparison between levels	Head	Wing
*Triatoma garciabesi*	Female	Low vs. high	0.93	1.84
		Low vs. intermediate	0.74	1.55*
		Intermediate vs. high	0.47	2.06
	Male	Low vs. high	0.68	2.51***
		Low vs. intermediate	1.81***	2.41***
		Intermediate vs. high	1.78*	2.36
*Triatoma guasayana*	Female	Low vs. high	0.35	1.29**
		Low vs. intermediate	0.85***	0.52
		Intermediate vs. high	0.73	1.08
	Male	Low vs. high	0.46	1.57**
		Low vs. intermediate	0.85*	1.27**
		Intermediate vs. high	0.69	1.10

Tests of 10,000 permutations

*P < 0.05

**P < 0.01

***P < 0.0001

Results of the pairwise comparison of head and wing shape across levels of anthropization are presented in Table 2. For *T. garciabesi* females, wing shape variation increased with the anthropization gradient, while for males, head shape increased with anthropization gradient (Table 2). For *T. guasayana,* males, variation in head shape decreased as the anthropization gradient increased (Table 2).

**Table 2:** Head and wing shape comparison between levels of anthropization for *Triatoma garciabesi* and *T. guasayana* populations

Species	Sex	Anthropization level	Procrustes variances	
			Module	
			Head	Wing
*Triatoma garciabesi*	Female	Low	0.71^a^	0.67^a^
		Intermediate	0.51^a^	0.89^b^
		High	0.84^a^	0.78^ab^
	Male	Low	0.49^a^	0.84^a^
		Intermediate	0.76^b^	0.67^a^
		High	0.60^ab^	0.77^a^
*Triatoma guasayana*	Female	Low	0.43^a^	0.85^a^
		Intermediate	0.44^a^	0.81^a^
		High	0.46^a^	0.79^a^
	Male	Low	0.65^a^	0.74^a^
		Intermediate	0.49^b^	0.79^a^
		High	0.37^b^	0.88^a^

For better visualization, the value of shape variance between anthropization levels is multiplied by 1000. Different letters indicate significant differences between comparisons

## Discussion

### Dispersal strategies along the anthropization gradient

The degradation of natural environments due to anthropogenic activities could indirectly affect triatomine populations, leading to their dispersal into artificial ecotopes [13]. Changes in the local environment, including decreases in bird and mammal populations as food sources and the loss of natural habitats, may exert selective pressures on flight-related traits that enhance mobility in dynamic environments [14, 15]. Our results showed that the density of insects invading dwellings differed between the two species in response to the anthropization gradient. While *T. garciabesi* showed higher density in landscapes with low levels of anthropization, *T. guasayana* showed similar densities across the levels of anthropization. These differences suggest that the two species have varying sensitivities to changes in population densities in landscapes experiencing frequent alterations [5]. Landscape anthropization has the potential to alter the structure of patches in unpredictable ways, reducing heterogeneity within patches while increasing it between them. The observed variation in landscape structure and heterogeneity may enhance the demographic stochasticity of populations and induce changes in their dispersal strategies [51]. *Triatoma garciabesi* can thrive in habitats with greater natural cover because of its specialized feeding habit of consuming birds. However, it is more susceptible to the loss of this cover due to human intervention in the landscape, likely because of its specialized feeding habit of consuming birds. In the context of landscape changes, birds are vulnerable to habitat loss caused by fragmentation [21]. In contrast, *T. guasayana* may be more resilient to landscape change because of its adaptation to more unstable environments (e.g. bromeliads, chimiles and dry logs) that are unable to buffer extreme weather conditions during winter. Additionally, *T. guasayana* exhibits a more generalist feeding habit, consuming both birds and mammals, with a preference for goats in the peridomestic environment [5–9]. Furthermore, this species has a dispersal strategy that is largely adapted to flight, allowing a greater number of individuals to move across the landscape and invade rural dwellings more frequently [6, 10, 11, 35]. Therefore, increased dispersal capacity allows this species to explore multiple environments until it finds the most suitable one for establishment and colony growth, thus providing greater resilience to the environmental pressures caused by anthropization.

Our results demonstrate shifts in the sex ratios of both species along the anthropization gradient. In landscapes with intermediate and high levels of anthropization, we observed declines in sexual dispersal for both species, with males experiencing greater declines than females. These findings suggest that selective pressures within patches may have differential effects on competition for resources, mates and inbreeding avoidance between the sexes, potentially resulting in altered dispersal dimorphism [52]. However, to comprehensively investigate this phenomenon, an experimental study exposing individuals to various pressures across multiple generations would be necessary to observe the long-term effects on dimorphism. Considering that females typically expend more energy due to oviposition, it is plausible that they exhibit greater movement, particularly given the likelihood of varying food availability across disturbance gradients. Therefore, we recommend a more nuanced approach and advocate for further research dedicated to addressing these questions.

### Flight dispersive attributes along the anthropization gradient in *T. garciabesi*

We found that *T. garciabesi* from less anthropized landscapes presented smaller body sizes than those from more anthropized landscapes. It has been suggested that the adult body size in insects is determined by the weight and duration of the last nymphal stage [53, 54]. Nutritional deficiencies during the last nymphal instar can lead to prolonged growth periods, resulting in larger adult sizes. It is possible that insects experience greater nutritional and environmental pressures in landscapes with increased anthropization, resulting in larger organisms, as human impacts on the habitat reduce the abundance and richness of bird and tree species that could provide food and shelter for *T. garciabesi* [16, 21].

Changes in wing morphology, including WL and WA, may indicate a selection process that maintains traits that improve flight performance for different landscape features [30]. In environments where the degree of anthropization increases, the wings of individuals exhibit higher WL and WA. This may facilitate greater deformation, resulting in increased lift and reduced energy expenditure during flapping [55, 56]. The narrow wing shapes ending in narrow tips may be associated with these morphological changes, allowing for longer straight flights when moving through the landscape matrix [15, 31, 32, 57].

Our results showed that changes in head size and shape showed sex differences which were more pronounced in males and related to more convex eyes in high levels of anthropogenic impacts. According to Hernández et al. [27, 28], macropterus individuals with greater compound eye convexity and higher EW might imply greater navigational ability and orientation during flight than micropterus. Conversely, sexual differences may indicate that males and females have different visual requirements for sexual dispersal behavior. In Lepidoptera, studies suggest that larger compound eyes in females may lead to increased ommatidial reallocation that enhances the search for oviposition sites, whereas in males the anterior region of the eye may play an important visual role in detecting and tracking flying females [58].

### Flight dispersive attributes along the anthropization gradient in *Triatoma guasayana*

No differences in mean body size were found across the anthropogenic gradient for either males or females. These results suggest that seasonality may play a more important role than anthropization gradients in the process of body size selection [38]. Ecotopes used by this species for shelters, including dry logs and bromeliads, have limited capacity to buffer external temperatures and humidity [7]. As a result, the frequency of small nymphs decreases and larger nymphs have a higher survival rate and thus metamorphose into large adults at the onset of the warm season [53, 54].

Wing size and shape of males and females differed across the anthropization gradient. Females showed no differences in wing size across the anthropogenic gradient. Males showed changes in the membranous wing region, with lower WL and WA in highly anthropized landscapes compared to those captured in low and intermediate anthropized landscapes. A reduction in the membranous region of the hemelytron could decrease the flexibility of the wing, which would negatively affect the production of aerodynamic forces, resulting in an increase in the energy cost required during flight [55, 59]. Our results showed that for both females and males, changes in mean wing shape were mostly observed in landscapes with less anthropization compared to landscapes with increasing anthropization. The average morphotype observed in landscapes with low levels of anthropization would be compatible with short, slow and maneuverable flights, allowing males to move through the environment while avoiding obstacles. In landscapes with intermediate and high levels of anthropization, a smaller wing insertion area could indicate greater speed during flight, and a thin distal tip could reduce the energetic cost of long-duration flights [31, 33, 57].

No changes in head size were observed across the anthropization gradient. However, differences in AD and EW were observed in males, which were high in the more anthropized landscapes. This may indicate a greater ability to adapt to environmental change in individuals that evolved in landscapes with high levels of anthropization. In contrast, shape changes recorded in both sexes were located at the level of the compound eye. In low and high anthropization landscapes, changes in eye configuration decreased convexity and shifted anteriorly, whereas in intermediate landscapes, eye convexity increased and shifted posteriorly. Eye convexity led to an increment in ommatidia distribution in the anterior region, favoring greater navigational ability in heterogeneous environments [28, 58].

### Both species responded differently to the anthropization gradient

Phenotypic changes were generally associated with the pressure of increased anthropization in both species. Our results showed that the response of both species was different. *Triatoma garciabesi* showed greater morphological changes than *T. guasayana*. Increasing anthropization gradients could exert more pressure on *T. garciabesi* by decreasing forest cover and increasing patch distances. Consequently, the number of dispersing individuals is reduced and selection on morphotypes compatible with rapid, less costly flights over greater distances increases. For both species, phenotypic changes were more pronounced in the males than in the females. Among triatomines, females are typically more dispersing than males during the reproductive period [10, 11, 35]. This dispersal behavior allows them to find and colonize new habitats, ensuring the survival of their offspring.

In this study, the techniques used to sample triatomines were only applied to dispersing individuals. To understand the relationship between environmental stress and phenotypic variation in triatomines, we need to compare the morphological changes and dispersal strategies of migrating and resident individuals in landscapes under increasing anthropogenic stress. In addition, the number of landscapes belonging to the different levels of anthropization was unbalanced. To improve the results, more landscapes with different levels of anthropization should be included. This would increase the number of replicates and improve the comparison between levels. In addition, further research is needed to better understand how triatomine species spread in their habitats and how they relate to human dwelling invasions.

## Conclusions

This study shows that the flight-dispersal characteristics of two triatomine species have changed in response to different levels of anthropization. Changes involve population densities, invasion frequency and differences in sex ratio across levels of anthropization; wing and head shape and size and variation of shape also varied across levels of anthropization. These findings may reflect divergent life strategies, adaptive capacity to different ecotopes, or a combination of both. Flight-mediated dynamics of house invasion, modified by habitat anthropization, may potentially favor the linkage of the wild *T. cruzi* transmission cycle to the peridomiciliary-domiciliary cycle.

### Supplementary Information

**Additional file 1: Table S1.** Description of landscape metrics extracted for each landscape in FRAGSTATS v4.2.

**Additional file 2:** **Table S2.** Confusion matrix for the map derived from the supervised classification analysis using Random Forest as the classifier. The diagonal values of the matrix represent the number of correctly classified pixels for each category, while the off-diagonal values indicate the number of incorrectly classified pixels. *UP* user’s precision, *PP* producer’s precision.

**Additional file 3: Table S3.** Comparison of inter- and intra-sex proportions within anthropization levels for *Triatoma garciabesi* and *T. guasayana*.

**Additional file 4: Table S4.** Results from analyzing body length, centroid size, head and wing linear measurements taken from adult specimens of *T. garciabesi* (**a**) and *T. guasayana* (**b**) that were gathered across landscapes exposed to three different degrees of anthropization (low, intermediate and high) are presented. Different letters indicate statistically significant differences seen between levels of anthropization for the same reference at *P* < 0.05 (Tukey’s post hoc tests for ANOVA; Dunn’s for Kruskal-Wallis as appropriate).

**Additional file 5: Table S5. **Reclassifying populations of wing and head shape variation in *Triatoma garciabesi* and *T. guasayana*. The analysis of canonical variables results in the number of accurately classified individuals.

**Additional file 6: Table S6.** Results were obtained through Mantel tests to examine the correlation between phenotypic plasticity and geographic distances. Multivariate regressions were conducted to determine the presence of allometry between the centroid size and shape component of the structures studied for both species and sexes. The correlation coefficient (r) was calculated. All tests were performed with 10,000 permutations.

## Data Availability

The datasets supporting the conclusions of this article are included in the article and its additional files. Raw data are available from the corresponding author on reasonable request.
