# Supplementary material for: Association between environmental gradient of anthropization and phenotypic plasticity in two species of triatomines
Source: Parasit Vectors. 2024 Apr 2;17:169. doi: 10.1186/s13071-024-06258-w (PMC10986143; doi:10.1186/s13071-024-06258-w)
Supplement: Supplementary file 1 — Additional file 1: Table S1. Description of landscape metrics extracted for each landscape in FRAGSTATS v4.2. [file 13071_2024_6258_MOESM1_ESM.docx]

**Additional file 1. Table S1.**

Description of landscape metrics extracted for each landscape in FRAGSTATS v4.2.

| Landscape metrics | Abbreviation | Description |
| --- | --- | --- |
| Landscape percentage | PLAND | Quantifies the proportional abundance of each patch type in the landscape. |
| Number of patches | NP | The quantity of patches belonging to a specific patch type serves as a straightforward indicator for the extent of division or fragmentation within said patch type. |
| Patch density | PD | Expresses the density of patches of a specific type per unit area. |
| Largest patch index | LPI | Determines the percentage of the overall landscape that constitutes the largest patch. |
| Landscape shape index | LSI | This provides a standard measure of the overall quantity of edge or edge density, corresponding to the scale of the landscape. |
| Average patch size | AREA_AM | The relationship between the number of patches within a class and the total area of that class. |
| Shape index distribution | SHAPE_AM | Measures the complexity of the patch shape relative to a standard (square) shape with the same dimensions. |
| Euclidean distance to nearest neighbor | ENN_AM | Quantifying patch isolation. |
| Landscape division index | DIVISION | The probability that two randomly selected pixels in a landscape do not belong to the same corresponding patch type is determined by a cumulative distribution of patch area. |
