# Supplementary material for: Association between environmental gradient of anthropization and phenotypic plasticity in two species of triatomines
Source: Parasit Vectors. 2024 Apr 2;17:169. doi: 10.1186/s13071-024-06258-w (PMC10986143; doi:10.1186/s13071-024-06258-w)
Supplement: Supplementary file 2 — Additional file 2: Table S2. Confusion matrix for the map derived from the supervised classification analysis using Random Forest as the classifier. The diagonal values of the matrix represent the number of correctly classified pixels for each category, while the off-diagonal values indicate the number of incorrectly classified pixels. UP user’s precision, PP producer’s precision. [file 13071_2024_6258_MOESM2_ESM.docx]

**Additional file 2. Table S2.**

Confusion matrix for the map derived from the supervised classification analysis using Random Forest as the classifier. The diagonal values of the matrix represent the number of correctly classified pixels for each category, while the off-diagonal values indicate the number of incorrectly classified pixels. UP: User's precision, and PP: Producer's precision.

| Classes | CF | OF | CS | OS | NVS | C | MG | W | Total | UP [%] |
| --- | --- | --- | --- | --- | --- | --- | --- | --- | --- | --- |
| Closed forest (CF) | 5933 | 0 | 0 | 2 | 0 | 0 | 0 | 0 | 5935 | 99.97 |
| Open forest (OF) | 0 | 23595 | 11 | 1 | 0 | 0 | 0 | 0 | 23607 | 99.95 |
| Closed shrubland (CS) | 0 | 40 | 9679 | 48 | 0 | 0 | 6 | 0 | 9773 | 99.04 |
| Open shrubland (OS) | 1 | 1 | 93 | 10704 | 4 | 0 | 67 | 0 | 10870 | 98.47 |
| Non-vegetated soil (NVS) | 0 | 0 | 0 | 5 | 9862 | 110 | 22 | 0 | 9999 | 98.63 |
| Crops (C) | 0 | 1 | 0 | 3 | 128 | 7826 | 85 | 0 | 8043 | 97.30 |
| Managed grassland (MG) | 0 | 4 | 7 | 15 | 12 | 11 | 28307 | 0 | 28356 | 99.83 |
| Water (W) | 0 | 0 | 0 | 0 | 0 | 0 | 0 | 1847 | 1847 | 100 |
| Total | 5934 | 23641 | 9790 | 10778 | 10006 | 7947 | 28487 | 1847 | 97753 |  |
| PP [%] | 99.98 | 99.81 | 98.87 | 99.31 | 98.56 | 98.48 | 99.37 | 100.00 |  |  |
| Global precision [%] | 99.31 |  |  |  |  |  |  |  |  |  |
| Kappa index | 0.992 |  |  |  |  |  |  |  |  |  |
