# Supplementary material for: Association between environmental gradient of anthropization and phenotypic plasticity in two species of triatomines
Source: Parasit Vectors. 2024 Apr 2;17:169. doi: 10.1186/s13071-024-06258-w (PMC10986143; doi:10.1186/s13071-024-06258-w)
Supplement: Supplementary file 3 — Additional file 3: Table S3. Comparison of inter- and intra-sex proportions within anthropization levels for Triatoma garciabesi and T. guasayana. [file 13071_2024_6258_MOESM3_ESM.docx]

**Additional file 3. Table S3**

Comparison of Inter- and Intra-Sex proportions within anthropization levels for *Triatoma garciabesi* and *T. guasayana*.

| Species | Comparison | Anthropization level | Number of individuals by sex and level of anthropization / Total number by level of anthropization (%) | χ^2^ | df | p-value |
| --- | --- | --- | --- | --- | --- | --- |
| *Triatoma garciabesi* | Intersexual | Low | Female: 24/56 (48.83)  Male: 32/56 (51.17) | 2.05 | 1 | 0.15 |
|  |  | Intermediate | Female: 25/34 (73.53)  Male: 9/34 (26.47) | 22.20 | 1 | <0.0001 |
|  |  | High | Female: 6/15 (40.00)  Male: 9/15 (60.00) | 4.00 | 1 | 0.09 |
|  | Intrasexual |  | Number of individuals by sex by level of anthropization / Total number by sex (%) |  |  |  |
|  | Female | Low | 24/55 (43.64) | 22.68 | 2 | <0.0001 |
|  |  | Intermediate | 25/55 (45.45) |  |  |  |
|  |  | High | 6/55 (10.91) |  |  |  |
|  | Male | Low | 32/50 (64.00) | 42.32 | 2 | <0.0001 |
|  |  | Intermediate | 9/50 (18.00) |  |  |  |
|  |  | High | 9/50 (18.00) |  |  |  |
| *Triatoma guasayana* | Intersexual | Low | Female: 81/119 (68.07)  Male: 38/119 (31.93) | 13.06 | 1 | 0.001 |
|  |  | Intermediate | Female: 105/151 (69.64)  Male: 46/151 (30.46) | 15.35 | 1 | <0.0001 |
|  |  | High | Female: 28/48 (58.33)  Male: 20/48 (41.67) | 2.78 | 1 | 0.09 |
|  | Intrasexual |  | Number of individuals by sex by level of anthropization / Total number by sex (%) |  |  |  |
|  | Female | Low | 81/214 (32.85) | 20.34 | 2 | <0.0001 |
|  |  | Intermediate | 105/214 (49.06) |  |  |  |
|  |  | High | 28/214 (13.08) |  |  |  |
|  | Male | Low | 38/104 (36.54) | 9.84 | 2 | 0.007 |
|  |  | Intermediate | 46/104 (44.23) |  |  |  |
|  |  | High | 20/104 (19.23) |  |  |  |
