# Supplementary material for: Association between environmental gradient of anthropization and phenotypic plasticity in two species of triatomines
Source: Parasit Vectors. 2024 Apr 2;17:169. doi: 10.1186/s13071-024-06258-w (PMC10986143; doi:10.1186/s13071-024-06258-w)
Supplement: Supplementary file 4 — Additional file 4: Table S4. Results from analyzing body length, centroid size, head and wing linear measurements taken from adult specimens of T. garciabesi (a) and T. guasayana (b) that were gathered across landscapes exposed to three different degrees of anthropization (low, intermediate and high) are presented. Different letters indicate statistically significant differences seen between levels of anthropization for the same reference at P < 0.05 (Tukey’s post hoc tests for ANOVA; Dunn’s for Kruskal-Wallis as appropriate). [file 13071_2024_6258_MOESM4_ESM.docx]

**Additional file 4. Table S4**

Results from analyzing body length, centroid size, head and wing linear measurements taken from adult specimens of *T. garciabesi* (a) and *T. guasayana* (b) that were gathered across landscapes exposed to three different degrees of anthropization (low, intermediate, and high) have been presented. Different letters indicate statistically significant differences seen between levels of anthropization for the same reference at p < 0.05 (Tukey's post-hoc tests for ANOVA; Dunn's for Kruskal-Wallis as appropriate).

| 1. *Triatoma garciabesi* | | | | | | | |
| --- | --- | --- | --- | --- | --- | --- | --- |
| Female | | | | | | | |
| Levels of anthropization | BL (mm) | Head | | | Wing | | |
|  |  | CS | AD (mm) | EW (mm) | CS | WL (mm) | WA (mm^2^) |
| Low | 18.38± 0.48^a^ | 2.07± 0.07^a^ | 1.85± 0.06^a^ | 1.88± 0.07^a^ | 10.02± 0.40^a^ | 8.49± 0.18^a^ | 27.39± 1.80^a^ |
| Intermediate | 18.80± 0.65^b^ | 2.08± 0.10^a^ | 1.90± 0.05^b^ | 1.95± 0.05^b^ | 10.20± 0.39^a^ | 8.72± 0.23^b^ | 29.15± 1.75^b^ |
| High | 19.22± 0.36^b^ | 2.07± 0.06^a^ | 1.91± 0.03^b^ | 1.94± 0.10^a,b^ | 10.07± 0.43^a^ | 8.75± 0.30^b^ | 28.74± 2.36^a,b^ |
| Male | | | | | | | |
| Levels of anthropization | BL (mm) | Head | | | Wing | | |
|  |  | CS | AD (mm) | EW (mm) | CS | WL (mm) | WA (mm^2^) |
| Low | 16.60± 0.60^a^ | 1.92± 0.05^a^ | 1.78± 0.04^a^ | 1.79± 0.07^a^ | 9.44± 0.47^a^ | 7.86± 0.23^a^ | 23.63± 1.14^a^ |
| Intermediate | 16.80±0.68^a,b^ | 2.01± 0.10^b^ | 1.81± 0.06^b^ | 1.81± 0.04^a^ | 9.60± 0.56^a^ | 8.22± 0.25^b^ | 24.92± 1.99^b^ |
| High | 17.25± 0.65^b^ | 1.98±0.05^a,b^ | 1.74±0.06^a,b^ | 1.82± 0.08^a^ | 9.46± 0.40^a^ | 8.07± 0.35^b^ | 24.30± 2.14^a,b^ |

| 1. *Triatoma guasayana* | | | | | | | |
| --- | --- | --- | --- | --- | --- | --- | --- |
| Female | | | | | | | |
| Levels of anthropization | BL (mm) | Head | | | Wing | | |
|  |  | CS | AD (mm) | EW (mm) | CS | WL (mm) | WA (mm^2^) |
| Low | 20.76± 0.86^a^ | 2.36± 0.09^a^ | 2.25± 0.08^a^ | 2.02± 0.08^a^ | 11.21± 0.47^a^ | 9.78± 0.40^a^ | 34.75± 2.82^a^ |
| Intermediate | 20.76± 0.91^a^ | 2.37± 0.09^a^ | 2.24± 0.07^a^ | 2.06± 0.09^a^ | 11.30± 0.55^a^ | 9.69± 0.45^a^ | 33.97± 3.28^a^ |
| High | 20.72± 0.88^a^ | 2.39± 0.09^a^ | 2.24± 0.09^a^ | 2.05± 0.09^a^ | 11.19± 0.45^a^ | 9.79± 0.39^a^ | 34.74± 2.71^a^ |
| Males | | | | | | | |
| Levels of anthropization | BL (mm) | Head | | | Wing | | |
|  |  | CS | AD (mm) | EW (mm) | CS | WL (mm) | WA (mm^2^) |
| Low | 18.66± 0.65^a^ | 2.22± 0.08^a^ | 2.09± 0.06^a^ | 1.93± 0.08^a,b^ | 10.37± 0.45^a^ | 9.04± 0.26^a,b^ | 29.77± 1.67^a,b^ |
| Intermediate | 18.93± 0.82^a^ | 2.22± 0.09^a^ | 2.10± 0.07^a^ | 1.96± 0.09^a^ | 10.41± 0.48^a^ | 9.11± 0.31^a^ | 30.31± 1.99^a^ |
| High | 18.63± 0.70^a^ | 2.20± 0.09^a^ | 2.05± 0.08^b^ | 1.89± 0.10^b^ | 10.23± 0.48^a^ | 8.83± 0.38^b^ | 28.41± 2.09^b^ |
