# Supplementary material for: Association between environmental gradient of anthropization and phenotypic plasticity in two species of triatomines
Source: Parasit Vectors. 2024 Apr 2;17:169. doi: 10.1186/s13071-024-06258-w (PMC10986143; doi:10.1186/s13071-024-06258-w)
Supplement: Supplementary file 5 — Additional file 5: Table S5. Reclassifying populations of wing and head shape variation in Triatoma garciabesi and T. guasayana. The analysis of canonical variables results in the number of accurately classified individuals. [file 13071_2024_6258_MOESM5_ESM.docx]

**Additional file 5. Table S5**

Reclassifying populations of wing and head shape Variation in *Triatoma garciabesi* and *T. guasayana*. The analysis of canonical variables results in the number of accurately classified individuals.

| Species | Sex | No. individual  n (%) | High  n (%) | Intermediate  n (%) | Low  n (%) | Module |
| --- | --- | --- | --- | --- | --- | --- |
| *Triatoma garciabesi* | Female | 31 (58) | 2 (33) | 13 (59) | 16 (64) | Head |
|  |  | 37 (70) | 3 (50) | 16 (70) | 18 (75) | Wing |
|  | Male | 35 (76) | 1 (14) | 5 (62) | 29 (93) | Head |
|  |  | 47 (94) | 9 (100) | 6 (67) | 32 (100) | Wing |
| *Triatoma guasayana* | Female | 124 (60) | 0 (0) | 83 (81) | 41 (55) | Head |
|  |  | 120 (57) | 7 (25) | 81 (78) | 32 (41) | Wing |
|  | Male | 59 (59) | 1 (5) | 38 (84) | 20 (57) | Head |
|  |  | 57 (56) | 7 (35) | 30 (67) | 20 (56) | Wing |
