# Supplementary material for: Association between environmental gradient of anthropization and phenotypic plasticity in two species of triatomines
Source: Parasit Vectors. 2024 Apr 2;17:169. doi: 10.1186/s13071-024-06258-w (PMC10986143; doi:10.1186/s13071-024-06258-w)
Supplement: Supplementary file 6 — Additional file 6: Table S6. Results were obtained through Mantel tests to examine the correlation between phenotypic plasticity and geographic distances. Multivariate regressions were conducted to determine the presence of allometry between the centroid size and shape component of the structures studied for both species and sexes. The correlation coefficient (r) was calculated. All tests were performed with 10,000 permutations. [file 13071_2024_6258_MOESM6_ESM.docx]

**Additional file 6. Table S6**

Results were obtained through Mantel tests to examine the correlation between phenotypic plasticity and geographic distances. Multivariate regressions were conducted to determine the presence of allometry between the centroid size and shape component of the structures studied for both species and sexes. The correlation coefficient (r) was calculated. All tests were performed with 10,000 permutations.

| Species | Sex | Module | Phenotypic plasticity associated with geographical distances | | Allometry | |
| --- | --- | --- | --- | --- | --- | --- |
|  |  |  | r | p-value | r | p-value |
| *Triatoma garciabesi* | Female | Head | 0.04 | 0.30 | 0.01 | 0.94 |
|  |  | Wing | 0.28 | 0.05 | 0.02 | 0.46 |
|  | Male | Head | 0.05 | 0.27 | 0.05 | 0.05 |
|  |  | Wing | -0.05 | 0.73 | 0.04 | 0.05 |
| *Triatoma guasayana* | Female | Head | -0.05 | 0.92 | 0.01 | 0.13 |
|  |  | Wing | 0.02 | 0.23 | 0.01 | 0.18 |
|  | Male | Head | 0.08 | 0.06 | 0.01 | 0.34 |
|  |  | Wing | -0.01 | 0.61 | 0.02 | 0.04 |
